# Does YouTube Provide Qualified Patient Education Videos About Atrial Fibrillation?

**DOI:** 10.3389/fpubh.2022.925691

**Published:** 2022-07-11

**Authors:** Chong Luo, Longrong Bian, Lijie Jiang, Weitao Liang, Zhong Wu

**Affiliations:** Department of Cardiovascular Surgery, West China Hospital, Sichuan University, Chengdu, China

**Keywords:** patient education, atrial fibrillation, We media, official account, physician, YouTube

## Abstract

**Objective:**

Patients utilize the internet as a pathway to acquire knowledge of specific diseases. However, there are limited oversight and review mechanisms to ensure the authenticity of online information. This study aimed to evaluate the quality of video-based resources used to obtain information about atrial fibrillation (AF).

**Methods:**

Multiple AF-specific keywords were used to perform a systematic search of YouTube. Two independent reviewers reviewed the top 50 results of each keyword search. To record data, the Journal of the American Medical Association (JAMA) score, modified DISCERN score, AF-specific score (AFSS), and essential score (Escore) were used. The Kruskal-Wallis test was used for intergroup comparisons.

**Results:**

A total of 74 videos that met the inclusion criteria were included in the analysis. In terms of video quality, 68% were poor, 19% were moderate, and 13% were exceptional. Videos submitted by publishers with a medical background were much less popular (*p* < 0.05) than those submitted by publishers without a medical background. The video quality did not differ among those included in this study.

**Conclusions:**

Some videos on YouTube that are of real value are not as popular as those with low-quality content submitted by news agencies/media publishers. Furthermore, videos submitted by those with a medical background do not receive as much attention as others. It is important to acknowledge that video platforms should establish content and quality auditing mechanisms for videos. Furthermore, publishers should ensure that viewers receive accurate and complete knowledge and use more concise and accessible images or animations that are tailored to the audience.

## Introduction

Atrial fibrillation (AF) is the most common clinical arrhythmia disorder. It affects approximately 33 million individuals worldwide (1) and has an estimated population prevalence of 2% to 4% (2). AF is usually followed-up and managed on an outpatient basis; however, the prolonged absence of patients from the care of physicians combined with inadequate recognition of the disease by some patients may result in adverse outcomes. The risk of ischemic stroke for patients with AF is much higher than that for patients without AF, and its consequences are severe, frequently recurrent, permanently disabling, and fatal (3, 4). Therefore, during the management of chronic diseases, such as AF, it is more important for patients to be fully aware of their pathophysiological status than for physicians to detail the dosage of medications and the timing of follow-up visits; however, both should be given full attention by physicians and patients.

Traditional health education has included the detailed explanation of illnesses by physicians at the clinic, distribution of educational materials such as pamphlets, and displaying of posters detailing relevant diseases; however, the streaming of videos on the internet has gradually become a contemporary and important way for patients and the general public to gain knowledge about diseases. YouTube is becoming an important source of non peer-reviewed medical information because it allows easy access to information and has worldwide popularity (5). The 2018 Health Information National Trends Survey mentioned that one-third of people have searched YouTube for videos about health-related topics (6). Although one study found that interns and attending physicians also acquire relevant knowledge by browsing online videos (7), the general population lacks knowledge of medical terminology, which can lead to the misunderstanding of non peer-reviewed audiovisual information. The anti-vaccine campaign on the internet, for example, prompted some individuals to refuse vaccination, thereby increasing the spread of infectious diseases and endangering the health of the entire population (8). This implies that the integration of online information obtained by such patients and the information communicated by their physicians can influence the process of making medical decisions. No study has investigated the availability and appropriateness of videos explaining AF as a patient-centered resource. In this study, we mimicked patients' self-searching behavior and systematically assessed the quality of videos posted on YouTube about the diagnosis and treatment of atrial fibrillation using four rating scales namely, Journal of the American Medical Association (JAMA), modified DISCERN, AF specific score, and essential score.

## Materials and Methods

### Study Design

This was an observational retrospective study. Using YouTube, we searched for the keywords “Atrial Fibrillation,” “AF,” and “AF+Management” on 13 September 2021, and recorded the URL, number of views, number of likes/dislikes, and number of comments/responses, which may change over time, of the videos. The video content and quality evaluations were completed within the following month.

### Measures

The keyword searches were performed by two authors using a cleared-cache web browser. They selected the top 50 results for each search term because patients who want to learn about AF (9, 10) are unlikely to exceed this range. Videos were excluded if they focused on only academic or narrative guidelines or diagnostic procedures. Videos relating to AF diagnosis, treatment, management were included in the analysis initially. Exclusion criteria were as follows: duplicate videos found during the search; videos with only audio; videos and video titles discordant with the content; and non-English language. Ultimately, 74 videos were included in the analysis.

### Data Collection

After a quick scan of the video during the first round of evaluation, the searcher checked whether the title and content of the video met the inclusion criteria. Finally, the two reviewers examined whether each of the videos met the standards. If there was a difference in opinion between the two reviewers, then it was resolved through consultation and re-examination. If no consensus was reached, then it was resolved by a third reviewer. The following data for each video were required: URL, video name, author qualification, upload date, video duration, number of views, numbers of likes, dislikes, comments, and author responses.

Because there is no scale that can be used to evaluate the quality of information provided by videos about AF, we developed a new scoring system, the AF specific score (AFSS), based on the existing literature and expert guidelines. The AFSS was found to have good reliability and validity (11, 12) (reliability: Cronbach's α=0.877; validity: Kaiser-Meyer-Olkin (KMO) = 0.785; and Bartlett's test of sphericity <0.001). The accuracy and reliability of the medical information in the retrieved videos were determined using the four standards of the JAMA score (13) and the modified DISCERN score created by Ubbink et al. (14) (Tables 1–3).

**Table 1:** JAMA Score benchmark criteria.

**Authorship:**	Authors and contributors, their affiliations, and relevant credentials should be provided.
**Attribution**:	References and sources for all content should be listed clearly, and all relevant copyright information noted
**Disclosure**:	Web site “ownership” should be prominently and fully disclosed, as should any sponsorship, advertising, underwriting, commercial funding arrangements or support, or potential conflicts of interest.
**Currency**:	Dates that content was posted and updated should be indicated.

*JAMA, Journal of American Medical Association*.

**Table 2:** Modified DISCERN criteria.

1. Are the aims clear and achieved?
2. Are reliable sources of information used? (ie, publication cited, speaker is board-certified vascular surgeon)
3. Is the information presented balanced and unbiased?
4. Are additional sources of information listed for patient reference?
5. Are areas of uncertainty mentioned?

**Table 3:** Video criteria grouped into parent categories.

**Diagnose**	**Treatment**	**Management**
1.Atrial Fibrillation Defination ***(Essential)***	1.Anticoagulant/Avoid stroke ***(Essential)***	1. Discussion of the indications/contradictions/possible complications of various treatments
2.Risk factors for AF ***(Essential)***	2. Surgical options such as AF ablation procedures(catheter/surgical)/left atrail appendage ***(Essential)***	2. Notifications of postoperative or drug follow-up care
3.Common clinical manifestation ***(Essential)***	3.Antiarrhythmic drug therapy ***(Essential)***	3.Mentions of AF is a disease that requires long-term treatment and needs patients' own participation in the management of this disease ***(Essential)***
4.Findings of a physical examination		4. Attention to the prevention and control of risk factors of AF
5.Electrocardiogram?Transthoracic echocardiography as diagnostic technique		
6.The consequences of allowing AF to be left untreated ***(Essential)***		

After the review, all videos were categorized into the following four groups according to whether the video publisher had a medical background: physicians group, medical facilities group, official accounts group, and news agencies/We media group (Table 4). The scoring criteria were not simple because some points required the existence of more than one item. For example, for AF, three treatment schemes are indispensable; if one happened to be missing, then it may cause patients to misunderstand their condition, which could then delay their seeking of a diagnosis and treatment by a physician. If the video that was watched by patients lacked the introduction of a surgical treatment plan, then it may have caused them to mistakenly think that their disease could be treated only with drugs, thereby causing them to have too high or too low expectations of the diagnosis and treatment. Furthermore, some patients could have received a late diagnosis because of their fear of surgical risks or serious complications induced by the videos. Based on the AFSS, we created the essential score (Escore) as the most basic scale for the diagnosis, treatment, and management of AF. When the AFSS and Escore conflicted, we were able to quickly find and re-evaluate the video quality. The maximum JAMA score, DISCERN score, and Escore were 4, 5, and 8, respectively; 1 point was deducted for each missing item, and the minimum score was 0 points. For the diagnosis section of the AFSS questionnaire, the maximum score was 5 points, and 1 point was deducted for each missing item; the minimum score was 0 points. In the management section of the AFSS questionnaire, the maximum score was 4 points, and 1 point was deducted for each missing item.

**Table 4:** Escore, AFSS, JAMA Score, and DISCERN values categorized by video publisher type on YouTube.

**Publisher type**	**N**	**Escore**	**AFSS**	**DISCERN**	**JAMA**	**Exceptional**	**Moderately**	**Poor**
Medical facilities	13	5.15 ± 2.48	6.08 ± 4.03	2.46 ± 0.66	1.92 ± 0.64	4	2	6
News agency/ We media	24	3.13 ± 2.23	3.46 ± 2.62	1.87 ± 0.74	1.42 ± 0.50	0	4	20
Official accounts	22	4.77 ± 2.67	5.73 ± 3.69	3.09 ± 1.07	2.95 ± 0.72	4	5	13
physician	15	3.87 ± 2.75	4.53 ± 4.61	2.87 ± 0.92	3 ± 0.66	2	3	11
Total	74	4.12 ± 2.60	4.81 ± 3.74	2.54 ± 1.00	2.28 ± 0.94	10	14	50
P-value	N/A	0.078	0.126	<0.001*	<0.001*	0.259	0.407	0.325

*Values are mean ± standard deviation (median).*

The * symbol indicates the statistical difference and the test method used in this analysis with a p value less than 0.05, Kruskal-Wallis test.

*The bold values indicates the p value which are less than 0.05 is considered to be statistically significant*.

These scoring criteria can determine whether the knowledge provided by the videos was sufficient. Furthermore, the essential item veto system excluded some videos that had sufficient elements but simply comprised a patchwork of knowledge points with little actual learning value. Therefore, this study was able to evaluate the usefulness and usability of videos through two screening mechanisms.

### Statistical Analysis

All videos were categorized into the physicians, medical facilities, news agencies/We media, and official accounts groups (Table 4) and rated as exceptional (10–13 points), moderately useful (5–9 points), or poor (0–4 points). Categorical variables are presented as frequencies and relative frequencies, and continuous variables are reported as means. The research results were statistically analyzed using IBM SPSS Statistics version 26 software. According to the Kolmogorov–Smirnov test, the data in this study were non-normally distributed. The Kruskal-Wallis test was used to perform comparisons between groups. The Mann-Whitney U test was used to distinguish significant differences between groups. *P* < 0.05 was considered significant. Theoretically, video publishers with a medical background should produce high-quality videos that are more popular among viewers because of their authority and production values. During this study, we compared the video durations and numbers of views, likes, comments, and responses by grouping videos according to their publisher type and quality.

## Results

**Figure 1:**
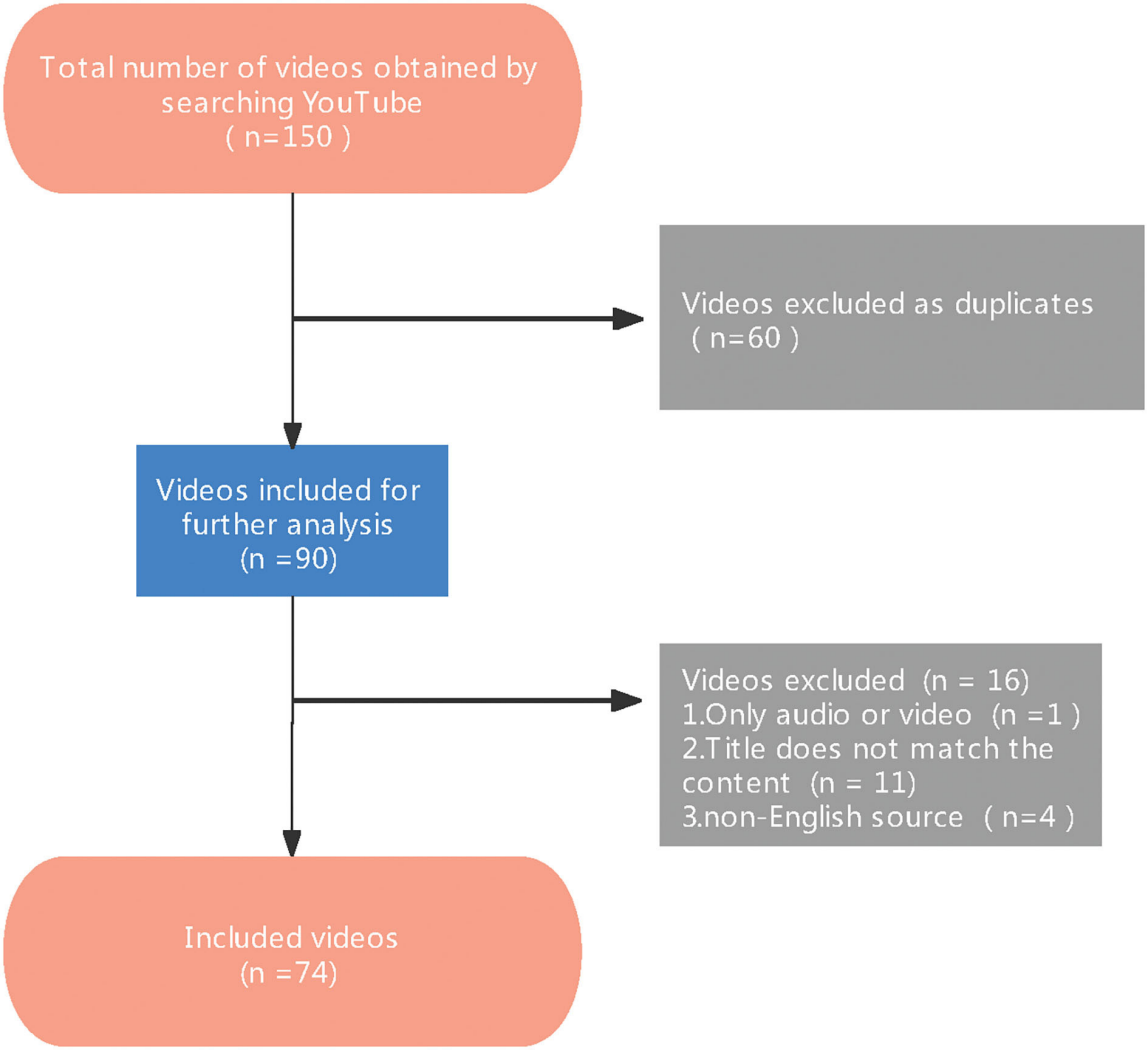
Flowchart demonstrating the process of video selection.

A total of 150 videos were included in the analysis. Of these, 60 videos were excluded because they were duplicates, four videos were excluded because they included non-English language, one video was excluded because there was no audio, and 11 videos were excluded because they were not relevant to the topic. Finally, 74 videos were selected for analysis (Figure 1); 16% of these videos were in the medical facilities group, 32% were in the news agencies/We media group, 30% were in the official accounts group, and 22% were in the physicians group.

**Figure 2:**
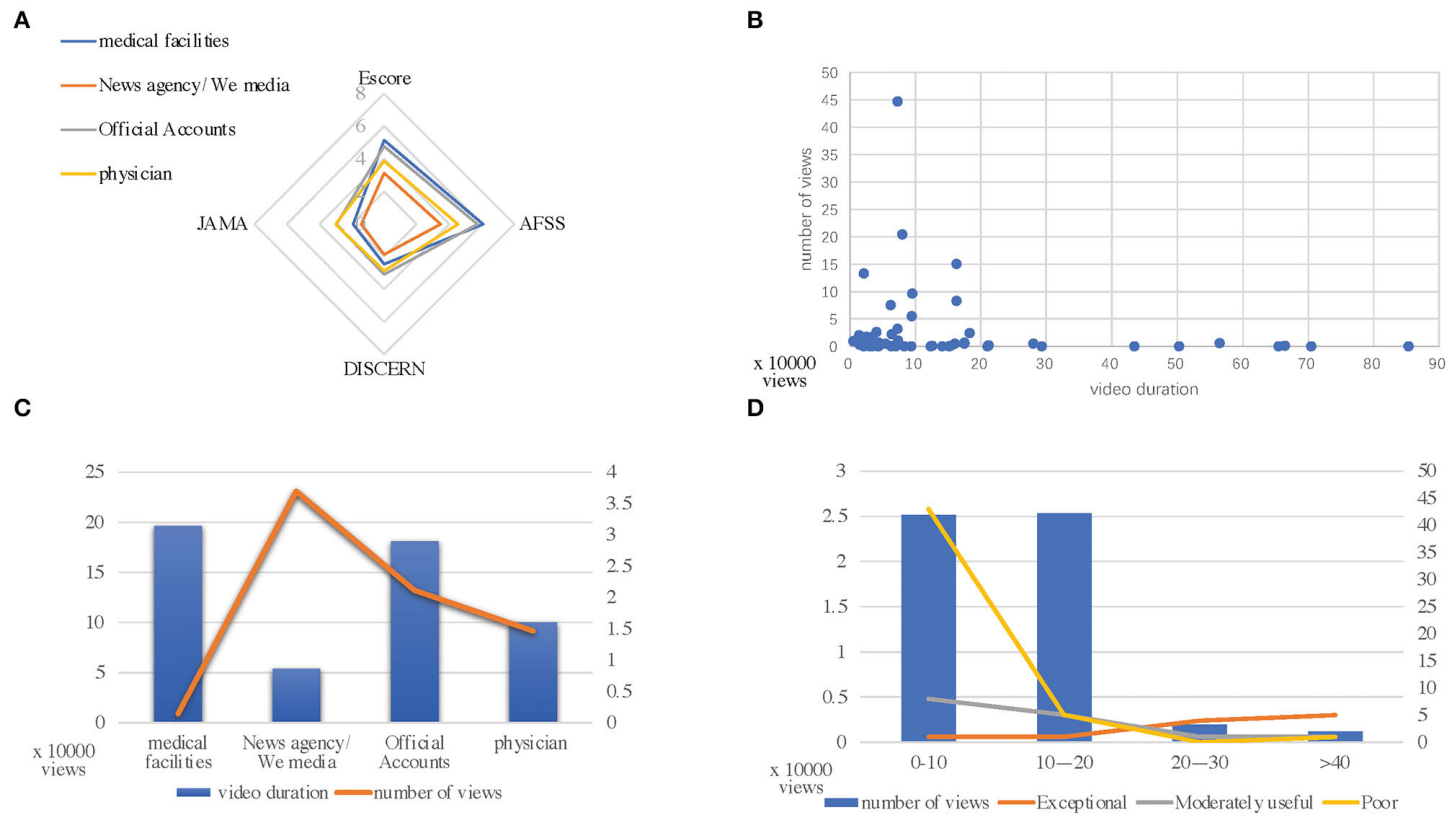
**(A)** Escore, Atrial Fibrillation Specific Score (AFSS), JAMA, DISCERN values categorized by video publisher type. **(B)** Relationship between views and video duration. **(C)** Relationship between views and publisher type. **(D)** Relationship between views and video quality.

The percentages of videos with exceptional and moderately poor quality were not statistically different (Table 4, Figure 2A). The median times of the videos in the medical facilities, news agencies/We media, official accounts, and physician groups were 19.65 min, 5.37 min, 18.10 min, and 10.00 min, respectively (*p* = 0.065). Videos in the news agencies/We media group had the highest number of views (>10,000) (p=0.003) and were mainly within 10 min in length. Only the number of likes was statistically different among groups (Table 5, Figures 2B,C).

**Table 5:** Objective data values categorized by video publisher type on YouTube.

**Publisher type**	**Mean. Video duration**	**Mean. Views**	**Mean. Likes**	**Mean. Dislikes**	**Mean. Comments**	**Mean. Author responses**
Medical facilities	18.69	1,428	15.75	0.5	1	0
News agency/ We media	7.79	36,963	479.75	12.1	20.63	2.29
Official Accounts	17.09	21,097	197.04	4.68	16.91	0.32
Physician	7.77	14,615	252.88	3.69	15.07	0.67
*p*-value	0.122	**0.003** ^*^	0.032*	0.074	0.234	0.128

The * symbol indicates the statistical difference and the test method used in this analysis with a p value less than 0.05, Kruskal-Wallis test.

*The bold values indicates the p value which are less than 0.05 is considered to be statistically significant*.

The JAMA score and modified DISCERN score were significantly higher for videos in the medical facilities and physician groups, which were published by those with a medical background, than for videos in the news agencies/We media and official accounts groups (*p* < 0.001). According to the video quality scores obtained using the Escore and the AFSS, there were a large number of low-quality videos with a duration of <10 min; furthermore, the number of views decreased as the running time increased (Figure 2D). Objective YouTube data (video duration, number of views, and number of likes) were obtained (*P* = 0.003). After integrating these data, we assessed the relationship between video quality and popularity and found that there was still a significant difference between the low-quality videos and medium-quality and high-quality videos during this study (*p* = 0.043) (Tables 5, 6). During this analysis, only the aforementioned indicators were significantly different.

**Table 6:** Objective data values categorized by video quality on YouTube.

**Video Quality**	**Mean. Video duration**	**Mean. Views**	**Mean. Likes**	**Mean. Dislikes**	**Mean. Comments**	**Mean. Author responses**
Exceptional	40.144	1,908	22.5	0.4	2.4	0
Moderately useful	14.6113	71,894	776	19.27	41.27	3.53
Poor	6.1639	10,301	127.32	3.37	9.63	0.39
*p*-value	<0.001*	0.043*	0.362	0.097	0.239	0.065

The * symbol indicates the statistical difference and the test method used in this analysis with a p value less than 0.05, Kruskal-Wallis test.

*The bold values indicates the p value which are less than 0.05 is considered to be statistically significant*.

## Discussion

This study aimed to provide a better understanding of the quality of evidence provided by videos independently accessed by patients with AF using a large online media-sharing platform (YouTube). We found that lower-quality and less comprehensive videos about AF available on YouTube are more popular. Furthermore, we found no significant differences in the content quality and number of comments when comparing video publishers with and without a medical background.

During our assessment of video quality and review of the previous literature, we found that Ferhatoglu et al. assessed the reliability, utility, and quality of sleeve gastrectomy video information and reported that the use of a single rating scale without a targeted rating system does not accurately assess the true value and accuracy of videos (15). Accordingly, we evaluated the video quality using the JAMA score and modified DISCERN score (13, 14); furthermore, we performed a routine assessment of video items because there is no scale that can evaluate the quality of information regarding AF and created the AFSS and Escore based on the available literature and expert guidelines (11, 12). Of the four rating systems, Escore, AFSS, DISCERN score, and JAMA score, only the DISCERN and JAMA scores (Table 4) were significantly different (*p* < 0.05) and able to reflect the rigor and structural integrity of the data used by the video publishers. The quality of the video content did not differ among these four rating systems, which was consistent with the results of Radonjic et al., who evaluated video information about abdominal aortic aneurysms. Theoretically, videos produced by physicians and authoritative medical institutions should have better structural integrity, data rigor, and video quality than those published by others; however, previous studies have not reached this conclusion (16–19). Additionally, our AFSS and Escore developed for AF did not differ significantly across the four subgroups of videos (Table 4). This seems to suggest that, on the YouTube platform, publishers with a medical background do not exhibit the expertise that they should. Our results were similar to those of Clerici et al., who analyzed a sample of 145 videos about rhabdomyosarcoma and reported that only 16.7% contained useful information and only one was posted by a physician and included correct information (20). In other words, if patients independently search the internet for knowledge regarding their AF symptoms, then they may find videos without rigorous data, with poor structural integrity, and with inadequate content.

The presentation of video content (graphic, animation, interview lecture, and conference video) varies based on the type of publisher. Gokcen et al. reported that YouTube videos of herniated disks presented by news agencies/We media and official accounts are usually designed to be more suitable for viewing by the general public (9). This has led to a higher number of views and increased popularity. During this study, we evaluated the relationship between the video publisher type and video quality and their associations with video popularity (Tables 5, 6). Interestingly, there was statistically significant variability in the number of video plays and the number of video likes; however, the other evaluated indicators were not significantly different. During our review, YouTube videos with AF content published by those with a medical background did not receive as much attention as videos published by those without a medical background (Figure 2C). These indicators suggest that there were differences in video popularity and that the number of post-view comments should increase proportionally to the number of video plays as the popularity increases; however, the gap between the number of views and the number of favorites was too large. We suspect that most viewers do not understand the content of the video and are unable to ask questions or make suggestions about the content, or that the site counts only those who click on the video as viewers, resulting in inflated view numbers. Studies have indicated that attempting to learn specific procedures, such as cardiopulmonary resuscitation, through self-learning videos is a viable approach (21). Therefore, it is important to ensure the accuracy of the content in these videos (22, 23). When we analyzed the relationship between video quality and the number of video views, we also found that lower-quality videos had significantly more views than higher-quality videos (*p* = 0.043) and that the differences among the numbers of views, comments, and replies were too large. Loeb et al. evaluated videos about bladder cancer on YouTube and reported the lack of a corresponding association between the number of video likes and video ratings, suggesting that most viewers do not effectively identify the quality of the videos (24). Similar results were obtained during this study. Using YouTube, we found that the mean video duration was within 10 min and that most of the videos with a high number of views (>10,000) were within this length (Figures 2B,C). However, there are several low-quality videos with durations of <10 min, and as the video duration increased, the quality of the videos improved, but the number of views decreased sharply (Figure 2D). This indicates that low-quality videos and videos with incomplete content are more easily retrieved and viewed. Regarding the viewers, they do not seem to be able to accurately identify the quality of the video based on views and likes, and they may like lower-quality videos, which seems to suggest that people who view a video and obtain correct but incomplete knowledge may have false perceptions of their illness. As a result, they may avoid seeking the advice of a physician and may not seriously consider their condition. Patients who are provided with misleading information and who have incorrect expectations will find it more challenging to seek medical guidance and the treatment they need.

With the rapid development of information technology, contemporary internet streaming video sites are becoming one of the most important ways for patients and the general public to gain knowledge about diseases. Because of its worldwide popularity (approximately two billion hits per day) and easy access to information by 95% of the world, YouTube is a great source of non peer-reviewed medical information (25). However, medical videos comprise a special category of videos that need to be standardized because they are related to human health; therefore, they should be reviewed before release. ReFaey et al. mentioned that the internet is an important platform for patients to access relevant resources; however, the control and censorship measures performed by these platforms for medical videos are limited and do not sufficiently ensure that the video content is accurate (26). It is evident that there are no standardized criteria for the intentions of the video publishers, video content, and audience demographics.

### Limitations

Although this study simulated the search behaviors of patients independently looking for data, it had some potential limitations. First, we used search terms that may not fully include what the general public might use. We used only the top 50 search results on the YouTube website and excluded non-English videos. Videos from other online platforms were not analyzed; therefore, we may have missed some of the videos that should have been included in the analysis. Objective evaluation indicators, such as the number of views, number of likes, and number of comments on online video sites are dynamic and may subsequently change or affect the statistical results. Second, the scales used for this study, although developed based on authoritative guidelines and the literature, were not professionally vetted. Third, the video search performed during this study only simulated patient behavior and may have been inaccurate. The quality of all videos on YouTube was not fully analyzed; therefore, the overall quality of the videos was not fully reflected.

## Conclusion

With the advent of information technology, people can access knowledge from multiple sources. This is a double-edged sword that can be both beneficial and harmful. Large amounts of video content regarding AF are available on the internet. During this study, we found that some YouTube videos that are of real value are not as popular as ones with low-quality content submitted by publishers without a medical background and that videos submitted by individuals with a medical background do not receive as much attention as others. The reason for this may be that the video content is not presented in a way that appeals to the masses, and that the quality does not differ from that of other publishers. It is essential to acknowledge that video platforms should establish auditing mechanisms for content and quality. Publishers should ensure that viewers receive accurate and complete knowledge while providing more concise and accessible images or animations tailored to the audience.

## Data Availability Statement

The original contributions presented in the study are included in the article/supplementary material, further inquiries can be directed to the corresponding author.

## Author Contributions

ZW and WL designed and revised the manuscript. CL, LB, and LJ performed data acquisition and statistical analysis. All authors have read and approved the final manuscript.

## Conflict of Interest

The authors declare that the research was conducted in the absence of any commercial or financial relationships that could be construed as a potential conflict of interest.

## Publisher's Note

All claims expressed in this article are solely those of the authors and do not necessarily represent those of their affiliated organizations, or those of the publisher, the editors and the reviewers. Any product that may be evaluated in this article, or claim that may be made by its manufacturer, is not guaranteed or endorsed by the publisher.

## Abbreviations

AF: atrial fibrillation
JAMA: Journal of the American Medical Association
AFSS: atrial fibrillation specific score
Escore: essential score
CPR: cardiopulmonary resuscitation.
